# Impact of COVID-19 restrictions on alcohol consumption behaviours

**DOI:** 10.1192/bjo.2021.986

**Published:** 2021-09-14

**Authors:** Emily O. C. Palmer, William Trender, Robin J. Tyacke, Adam Hampshire, Anne Lingford-Hughes

**Affiliations:** Division of Psychiatry, Department of Brain Science, Imperial College London, UK; The Computational, Cognitive and Clinical Neuroimaging Laboratory, Imperial College London, UK; Division of Psychiatry, Department of Brain Science, Imperial College London, UK; The Computational, Cognitive and Clinical Neuroimaging Laboratory, Imperial College London, UK; Division of Psychiatry, Department of Brain Science, Imperial College London, UK

**Keywords:** Alcohol disorders, cognitive neuroscience, psychosocial interventions, drugs of dependence disorders, epidemiology

## Abstract

**Background:**

We aimed to evaluate how coronavirus (COVID-19) restrictions had altered individual's drinking behaviours, including consumption, hangover experiences, and motivations to drink, and changing levels of depression and anxiety.

**Method:**

We conducted an online cross-sectional self-report survey. Whole group analysis compared pre- versus post-COVID restrictions. A correlation coefficient matrix evaluated the associations between all outcome scores. Self-report data was compared with Alcohol Use Disorders Identification Test (AUDIT) scores from the 2014 Adult Psychiatric Morbidity Survey. Multiple linear modelling (MLM) was calculated to identify factors associated with increasing AUDIT scores and post-restriction AUDIT scores.

**Results:**

In total, 346 individuals completed the survey, of which 336 reported drinking and were therefore analysed. After COVID-19 restrictions 23.2% of respondents reported an increased AUDIT score, and 60.1% a decreased score. AUDIT score change was positively correlated with change in depression (*P <* 0.01, *r =* 0.15), anxiety (*P <* 0.01, *r =* 0.15) and drinking to cope scores (*P <* 0.0001, *r =* 0.35). MLM revealed that higher AUDIT scores were associated with age, mental illness, lack of a garden, self-employed or furloughed individuals, a confirmed COVID-19 diagnosis and smoking status.

**Conclusions:**

COVID-19 restrictions decreased alcohol consumption for the majority of individuals in this study. However, a small proportion increased their consumption; this related to drinking to cope and increased depression and anxiety.

## Background

Coronavirus (COVID-19) has changed social behaviours on a global scale. A recent report from the Office of National Statistics reported that 37.4% of adults in Great Britain said the COVID-19 pandemic has affected their well-being.^1^ Indeed, a recent longitudinal study found that mental health problems assessed using the General Health Questionnaire-12 (GHQ-12) have increased substantially (from 23.4% in 2017–2019 to 37.1% in April 2020).^2^ In addition to growing mental health concerns, others anticipated that the wider implications of the COVID-19 pandemic will lead to an increase in alcohol and substance use disorders.^3,4^ For instance, recent data from Belgium suggests that COVID-19 lockdown measures resulted in increased consumption of both alcohol and tobacco^5^ and data from Public Health England and analysed by the Royal College of Psychiatry found that over 8.4 million people are now drinking amounts that put them at higher risk of ill health (assessed using a survey enquiring about units consumed in a typical week) compared with 4.8 million in February 2020.^6^ Similarly, a recent commentary noted that across a dozen studies 20–40% of surveyed participants reported drinking more.^7^

Such increased consumption may also adversely have an impact on vulnerability to COVID-19 as there is evidence that alcohol may disrupt the immune response.^8^ In addition, increased consumption is likely to translate to increased alcohol-specific deaths. Indeed, the Office of National Statistics reported that 2020 had the highest annual total of alcohol-specific deaths since the start of the data-series in 2001 (accessed June 2021).^9^ Indeed, deaths increased 19.6% compared with 2019 and have been increasing with every successive quarter. Further investigation is therefore required to understand the interplay between COVID-19 and alcohol consumption.

It has previously been shown that drinking motivations can be altered in response to the context of social facilitation.^10^ Drinking motivations have been linked to risk of developing alcohol use disorder.^11^ COVID restrictions have not affected all individuals equally,^12^ therefore certain characteristics may be related to increased alcohol consumption among some individuals. It is therefore critical to evaluate the relationship between drinking motivations, individual characteristics, mental health and increased alcohol consumption in response to the COVID-19 pandemic.

## Aims

We developed a self-report survey with the aim of understanding how pandemic-related restrictions altered alcohol consumption, motivations to drink as well as depression and anxiety. We hypothesised that COVID-19 restrictions would increase overall alcohol consumption, and that this would be associated with an increase in the motivation to drink to cope, and anxiety and depression scores. We predicted that because of the removal of social drinking occasions there would be a reduction in binge drinking (six or more drinks on one drinking occasion) occurrences, but that individuals would be drinking more regularly.

## Method

### Participants

Participants were recruited between 4 June and 5 August 2020 via social media platforms (Instagram, Facebook and Twitter). Participants could complete the survey if they had internet access and were English speaking. Participants younger than 16 years old were shown a message indicating some parts of the survey may be irrelevant to them but they were allowed to participate (for example financial security questions). However, no respondents were 16 years old or younger. Prior to completing the survey participants were required to provide informed consent (by ticking boxes online) to participate in the survey. All data was collected anonymously.

### Procedures

The survey was launched and run from the hangover.cognitron.co.uk website. Participants completed a single survey divided into three sections: background, pre-COVID-19 and post-COVID-19. The background section collected demographic information, employment status, country and details of current implemented COVID-19 restrictions and financial security. Participants were instructed to answer questions regarding their emotions and behaviours before the implementation of COVID-19 restrictions (based on their recall) followed by the same questions after the implementation of COVID-19 restrictions (current emotions and behaviours). The participant determined which COVID-19 restrictions they were referring to rather than specify a particular date or action because of the multiplicity of different approaches experienced by individuals in various countries. Participants were then asked, ‘In this section of the survey please respond with what is correct for you AFTER you started following the COVID-19 related restrictions you have previously described’ (henceforth referred to as ‘COVID-19 restrictions’). The survey was designed to take about 20–25 min to complete (on average it took 13 min). Participants were able to stop taking part at any stage, only completed survey data was collected.

Approval was obtained from Imperial College Research Ethics Committee, reference: 20IC5961. The study was conducted in accordance with the recommendations for research on human participants adopted by the 18th World Medical Assembly, Helsinki 1964 and later revisions.

### Measures

The Alcohol Use Disorders Identification Test (AUDIT) was used to evaluate alcohol consumption, drinking behaviours and alcohol-related problems.^13,14^ The self-report nine-item Patient Health Questionnaire (PHQ-9) and seven item Generalized Anxiety Disorder (GAD-7) assessed depression and anxiety, respectively.^15,16^ For both measures scores of 5, 10, 15 and 20 represent mild, moderate, moderately severe and severe thresholds.^15–17^

The 20-item Drinking Motives Questionnaire, Revised (DMQ-R) assessed the participant's motives to consume alcohol using a 1–5 scale from ‘almost never/never’ to ‘almost always/always’.^18^ This questionnaire has four subscales: social, coping, conformity and enhancement. To determine sensitivity to hangovers, participants were asked ‘Do you experience hangovers?’ requiring a simple yes/no response. We developed a Hangover Questionnaire to evaluate drinking behaviours that induce a hangover (Supplementary Material 1 available at https://doi.org/10.1192/bjo.2021.986).

Participants were asked to rate their concern about COVID-19 over the past 2 weeks compared with before the COVID-19 restrictions (Supplementary Material 2). This gave a total COVID concern score for which the maximum was 48, and 24 indicated no change from normal.

### Analysis

All analyses were completed using SPSS (v25, 2017) and Prism (v9, 2020). Prior to analysis all variables were tested for normality. Several of the data-sets were not normally distributed and therefore non-parametric tests were used for the analysis. The outcome score ‘change’ refers to the difference in self-report scores pre- versus post-COVID-19 restrictions. The Wilcoxon signed-rank test was used to compare whole group pre-restriction data with post-restriction data. Spearman rank correlation coefficient matrix was used to evaluate the strength of associations between all outcome scores with the significance cut-off of *P* < 0.05. Given the possible effects of recall bias the AUDIT scores from our participants were compared with previous population results from the publicly available data from the Adult Psychiatric Morbidity Survey (APMS).^19^

Multiple linear modelling (MLM) tested how cohort demographics and pandemic specific factors (such as age, gender, concern about finances because of the pandemic) were associated with change in AUDIT scores and post-restriction AUDIT scores. We looked at both AUDIT change and post-restriction AUDIT scores to account for a potential effect of recall bias. To help identify if the direction of change in drinking consumptions (increase versus decrease) was influenced by the pre-existing drinking behaviours an ANCOVA with *post hoc* multiple comparisons was used to mitigate the effect of regression to the mean (RTM). All figures are given as mean (s.d.) unless otherwise specified.

## Results

In total, 346 participants responded to the survey, of which 10 were teetotal so their data was not analysed further leaving data from 336 participants. All demographic data and COVID-19-related participant characteristics are shown in Table 1. The majority of respondents were female (64.3%), between age of 21 and 40 (85.8%), White (90.5%), residence in the UK (75.6%) in a relationship (41.7%) and achieved an undergraduate or postgraduate degree (84.9%).

**Table 1:** Participants characteristics

Characteristics	*n*	%
Age, years		
18–20	7	2.1
21–25	85	30.1
26–30	80	28.4
31–40	77	27.3
41–50	38	13.5
51–60	24	8.5
>60	24	8.9
Gender		
Male	116	34.5
Female	216	64.3
Other	3	0.9
Prefer not to say	1	0.3
Ethnicity		
White	304	90.5
Other ethnic background	32	9.5
Marital status		
Married	97	28.9
In a relationship	140	41.7
Single	96	28.6
Other	2	0.6
Highest educational level		
School	14	4.2
College	33	9.8
Undergraduate degree	146	43.5
Master's degree (Or equivalent)	99	29.5
PhD	40	11.9
Other	4	1.2
Alcohol dependence (self-report not from AUDIT)		
Currently dependent	11	3.3
Previously dependent	11	3.3
No history of dependence	312	92.9
Other	2	0.6
Illicit drug dependence (self-report)		
Currently dependent	5	1.5
Previously dependent	15	4.5
No history of dependence	312	92.9
Other	4	1.2
Smoking (tobacco) status		
Smoker	65	19.3
Non-smoker	271	80.7
Country of residence		
UK	254	75.6
USA	46	13.7
Other	36	10.7
Residential area		
City	187	55.7
Suburban	92	27.4
Rural	57	17.0
Residence type		
Flat or apartment	125	37.2
House without garden	16	4.8
House with garden	189	56.3
Other	6	1.8
Number of people in residence		
1	44	13.1
2	129	38.4
3	56	16.7
4 or more	107	31.8

As a group, AUDIT scores were significantly lower after COVID-19 restrictions and fell below the threshold considered ‘harmful or hazardous’ (Table 2). Interestingly, COVID-19 restrictions increased scores for anxiety and depression to above the respective ‘mild’ diagnosis thresholds (GAD-7 > 5, PHQ-9 > 5). Almost a quarter (23.2%) of respondents reported an increase in AUDIT score pre- to post-COVID-19, but 60.1% reported a decreased score.

**Table 2:** Measures pre- versus post-COVID-19 restrictions^a^

	Pre-restriction, mean (s.d.)	Post-restriction, mean (s.d.)	*Z*	*P*	Direction of change
GAD-7 score	4.5 (4.7)	6.3 (5.5)	−8.2	<0.0001	Increased
PHQ-9 score	3.7 (4.3))	6.6 (5.8)	−11.2	<0.0001	Increased
AUDIT score	8.6 (5.3)	6.7 (5.6)	−7.8	<0.0001	Decreased
Consumption frequency score	2.8 (0.8)	2.7 (1.12)	−1.9	0.053	Decreased
Number of drinks score	0.9 (1.0)	0.7 (0.9)	−3.7	<0.0001	Decreased
Binge frequency score	1.5 (1.1)	1.1 (1.2)	−6.3	<0.0001	Decreased
Hangover frequency	2.7 (1.8)	2.4 (2.2)	−3.6	<0.0001	Decreased
Hangover sensitivity	1.6 (1.0)	1.2 (1.2)	−5.9	<0.0001	Decreased
DMQ: social	14.7 (5.2)	10.2 (5.2)	−13.1	<0.0001	Decreased
DMQ: coping	9.0 (3.8)	9.2 (4.6)	−0.2	0.875	Increased
DMQ: enhancement	13.2 (5.0)	12.2 (5.3)	−4.8	<0.0001	Decreased
DMQ: conformity	7.3 (3.0)	6.1 (2.6)	−8.6	<0.0001	Decreased

GAD, Generalized Anxiety Disorder; PHQ, Patient Health Questionnaire; AUDIT, Alcohol Use Disorder Test (AUDIT); DMQ, Drinking Motives Questionnaire.

a. Comparisons between individual scores analysed using Wilcoxon signed-rank test statistics.

Of the individual factors of the AUDIT, quantity of alcohol consumed and binge drinking were significantly decreased, but the frequency of consumption remained unchanged pre- versus post-COVID restrictions (Table 2). Further, respondents reported that hangover frequency and the number of drinks required to induce a hangover was significantly reduced. As shown in Table 2, all the drinking motives were significantly decreased except ‘Coping’ which remained the same.

The AUDIT scores from our participants were compared with previous national population results (with non-drinkers removed) from the APMS (Supplementary Material 3).^20^ The pre-restriction self-report AUDIT scores suggest a significantly higher proportion of hazardous and harmful and dependant drinkers (38%, 10% and 5%, respectively) when compared with APMS results (22%, 2% and 2%, respectively). However, when the APMS data is compared with the proportion of hazardous and harmful and dependant drinkers reported in the post-restriction AUDIT scores (21%, 4% and 6%, respectively) they are in close agreement.

**Fig. 1:**
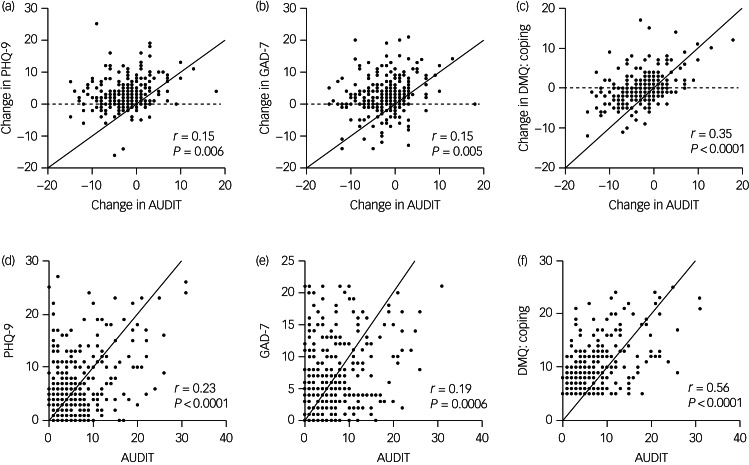
Correlation of change in (a) anxiety, (b) depression and (c) motivation to drink to cope with change in scores of Alcohol Use Disorders Identification Test (AUDIT) given the impact on coronavirus disease 2019 (COVID-19) restrictions. Current (during the pandemic, post-COVID restriction implementation) self-report scores for (d) anxiety, (e) depression and (f) motivation to drink to cope correlated with current AUDIT scores. Correlation analysis used Spearman's rank correlations. GAD, Generalized anxiety disorder; PHQ, Patient Health Questionnaire, DMQ, Drinking Motives Questionnaire.

The strength of associations between drinking behaviour, motivations to drink, and anxiety and depression scores were evaluated using Spearman rank correlation analysis Fig. 1(a and b). Figure 1(a) illustrates significant positive correlations between the change in AUDIT score and the change in depression (*r* *=* 0.15, *P* *<* 0.01), anxiety (*r* *=* 0.15, *P* *<* 0.01) and drinking to cope scores (*r* *=* 0.35, *P* *<* 0.0001). To diminish risk of recall bias, we also present correlations using self-report post-restriction scores. As shown in Fig. 1(b) there is a positive correlation between post-restriction AUDIT scores such that as they increase so to do the post-restriction scores for: depression (*r* *=* 0.23, *P* *<* 0.0001), anxiety (*r* *=* 0.19, *P* *<* 0.001) and drinking to cope (*r* *=* 0.56, *P* *<* 0.0001).

**Fig. 2:**
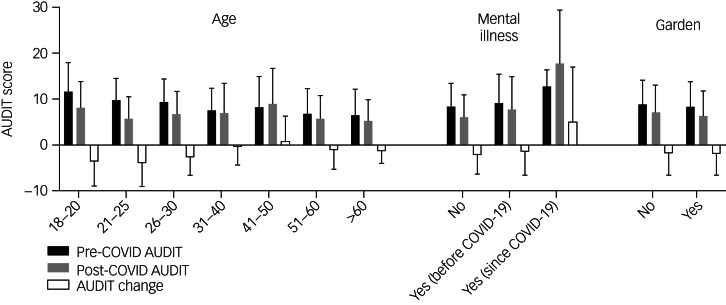
Impact of cohort factors on pre- (black), post- (grey) and change (white) in scores of Alcohol Use Disorders Identification Test (AUDIT) since the implementation of coronavirus disease 2019 (COVID-19) restrictions. Data collected from 336 survey participants. Data shown as mean (s.d.).

We used MLM to test how cohort characteristics (for example age, gender, concern about finances because of the pandemic) were associated with change in AUDIT scores and post-restriction AUDIT scores. Each cohort factor was considered while accounting for the influence of all the other factors. Characteristics that significantly had an impact on both the change in AUDIT scores and post-restriction AUDIT scores are illustrated in Fig. 2. Both an increase in AUDIT score and higher post-restriction AUDIT score were associated with the age group 41–50 (|*t*| = 3.648, *P* *<* 0.00, |*t*| = 2.023, *P* *<* 0.05, respectively, where *t* is the *t* statistic of the multiple linear regression), participants that reported they had received a diagnosis of mental illness since the start of the COVID-19 restrictions (|*t*| = 3.163, *P* *<* 0.01, |*t*| = 3.963, *P* < 0.0001, respectively) and participants living in a residence with no garden (|*t*| = 3.163, *P* *<* 0.01, |*t*| = 2.096, *P* < 0.05, respectively).

Additionally, an increase in AUDIT score was associated with self-employed currently working individuals, furloughed individuals, or individuals with a confirmed COVID-19 diagnosis (|*t*| = 2.159, *P* *<* 0.05, |*t*| = 2.551, *P* *<* 0.05, |*t*| = 2.386, *P* *<* 0.05, respectively). However, occupation and COVID-19 diagnosis were not associated with post-restriction AUDIT score. Conversely, smoking status was associated with increase post-restriction AUDIT score (|*t*| = 4.467, *P* *<* 0.0001) but not AUDIT change.

**Fig. 3:**
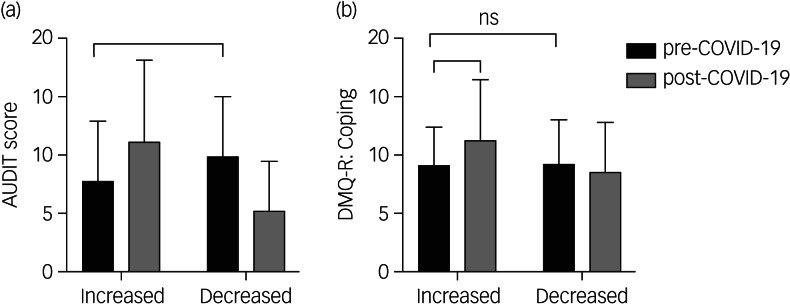
Comparison of Alcohol Use Disorders Identification Test (AUDIT) (a) and drinking to cope (b) scores between individuals who increased or decreased alcohol consumption since implementation of coronavirus disease 2019 (COVID-19) restrictions. For each group (increased versus decreased) self-report scores are shown pre-COVID-19 (black) and post-COVID-19 (grey) restrictions. Data presented is mean (s.d.) of 336 survey participants. *Significant at *P* < 0.05; ns, non-significance.

We undertook a one-way ANCOVA on data for the individuals whose AUDIT score had increased compared with those whose drinking had decreased with implementation of COVID-19 restrictions, while controlling for post- restriction AUDIT score to mitigate the effect of RTM (Fig. 3(a)). There was a significant effect of group (increased versus decreased) on pre-restriction AUDIT score (*F*(2,332) = 119, *P* *<* 0.0001). However, of particular interest was the result of the *post hoc* tests that revealed that those in the group who increased their AUDIT score had a significantly lower average AUDIT score before the COVID-19 restrictions, than individuals who decreased their AUDIT score (*P* *<* 0.0001).

A two-way ANOVA was used to assess if ‘coping’ as a motivation to drink differed between those whose AUDIT score had increased compared with those in whom it decreased (Fig. 3(b)). As expected, we found a significant effect of group (increased versus decreased) (*F*(1, 536) = 11.11, *P* < 0.001) and restriction (pre- versus post-COVID-19) (*F*(1, 536) = 4.21, *P* *<* 0.05). The *post hoc* Tukey's multiple comparison test revealed no significant difference in coping scores before the COVID- 19 restrictions between individuals who had an increased versus a decreased AUDIT score (*P* = 0.99). However, as expected post-COVID-19 restriction coping score was significantly higher compared with pre-COVID-19 restriction coping score in those in the group who increased their AUDIT score (*P* *<* 0.01).

Finally, we looked at the characteristics of those whose AUDIT score had increased with those whose score had decreased (Supplementary Material 4). The ratio of males and females were similar in both groups (increased: female 62.8%, male 35.9% *v.* decreased: female 65.1%, male 33.9%). Interestingly, a higher proportion of individuals with a previous history of alcohol dependence were in the increased group (10.3%) compared with the decreased group (3.6%). Regarding age, AUDIT scores decreased largely in younger participants whereas all other ages were associated with an increased score.

## Discussion

### Main findings

We found that although COVID-19 restrictions have overall reduced AUDIT scores within our cohort, almost a quarter (23.2%) reported an increase. We found that there were significant positive correlations between anxiety and depression scores and increasing AUDIT scores, consistent with recent evidence that found alcohol intake was associated with higher depression, anxiety and stress symptoms.^21–23^ The largest increase in consumption was seen in those who reported increased ‘drinking to cope’ as a motivation. Notably, this ‘coping’ motivation also correlated with both increased anxiety and depression scores because of COVID-19 restrictions. With regards to alcohol consumption, participants did not report a change in how often they were consuming alcohol but did report a significant reduction in the number of drinks and a reduction in binge drinking (six or more drinks on one drinking occasion).

### Interpretation of our findings

When comparing the pre-restriction AUDIT scores of those individuals who increased versus decreased their drinking, those individuals whose AUDIT score decreased, initially consumed more alcohol than those whose score increased. Those individuals who had an increased AUDIT score also had a significant increase in their motivation to drink to cope score compared with those that had a decreased AUDIT score. This is consistent with the self-medication hypothesis^24,25^ and shows that COVID-19 restrictions have led to an increase in coping-motivated drinking, within these individuals. Of concern was that a higher proportion of participants with a self-reported history of alcohol dependence had an increased AUDIT score after the implementation of COVID-19 restrictions that likely reflects relapse. Future studies should evaluate if the pandemic is leading to higher relapse rates in recovering alcoholics.

A range of other factors were associated with increased AUDIT scores including age, a diagnosis of mental illness since the onset of the pandemic, currently working self-employed or furloughed staff, a confirmed positive COVID-19 diagnosis, smoking status and living in a residence without a garden. Notably most participants whose AUDIT score decreased were in the younger age groups, likely because of reduced social activities. Indeed, we attribute the disparity between our pre-restriction self-report AUDIT scores and the population data from the 2014 APMS to our participants being largely from younger age groups.

We found that those currently working self-employed or furloughed staff was associated with increased consumption. This is unsurprising as these groups are the most likely to experience the largest financial/job- related concern. It has been previously shown that low job control and high demands at work are related to higher levels of drinking to cope.^26^ Additionally, studies have shown that workers who felt their skills were underused and/or were not involved in decision-making, as many furloughed staff must have experienced, had higher levels of heavy drinking.^27^ Furthermore, the association we found between a diagnosis of mental illness since the onset of the pandemic or a confirmed positive COVID-19 diagnosis with increased AUDIT score may be explained also by the self-medication hypothesis to alleviate anxiety and stress.^28,29^

Our respondents were predominantly female though we found no association between gender and AUDIT change or post-AUDIT score. This differs from other studies that found that COVID-19 psychological distress resulted in increased drinking only in females.^29,30^ This may be because of the younger average age of our cohort (58.5% aged 21–30) compared with the average age of the previous study (41.7 years). Future research should evaluate the effect of age in drinking-related gender differences in response to psychological distress. Although we found that AUDIT scores overall were reduced in our cohort, scores did increase in a substantial minority. Other surveys have reported an increase in consumption of 20–30% in UK, Australia and Belgium as a result of COVID restrictions.^5,6,21,30–32^ However, our finding of an overall reduction in alcohol consumption is supported by an Australian study that reported the lowest levels of the alcohol metabolite ethyl sulphate in wastewater in April 2020 compared with previous years.^33^

### Limitations

The cross-sectional self-report nature of the study is a major limitation as it is subject to reporting, self-perception and recall bias. We attempted to mitigate recall bias by analysing both perceived change in outcome scores and post-restriction scores (as current behaviour should be less effected by recall bias). We are also cautious about inferring that the increased self-report scores relate to increases in clinically significant levels of anxiety and depression. Further, we acknowledge that the AUDIT is designed as a screening instrument for recent alcohol-related behaviours rather than to assess previous behaviours, however, we felt it was the best instrument to use to compare our study with other studies.

We acknowledge our relatively small sample size and our online sampling strategy are key limitations of this study. Additionally, the lack of ethnic diversity and the high education level of respondents means that findings are not necessarily generalisable to the general population. Given the increased risk COVID-19 presents to those from Black and ethnic minority backgrounds,^34^ future studies should assess changes in alcohol consumption in these individuals. Future studies should aim to assess how COVID-19 restrictions altered drinking behaviours in a larger and more representative sample. Additionally, caution should be taken when interpreting comparisons with the AMPS data-set as the AMPS only included UK residents whereas our study did not. Similarly, the AMPS had a much larger more diverse cohort and employed more stringent recruitment than the current study.

### Implications

Restrictions to reduce the spread of COVID-19 significantly reduced overall AUDIT scores. This reduction was driven by a decrease in the quantity of alcohol consumption, whereas the frequency remained unchanged. Our data suggests this is likely the result of a reduction in social and societal norms that encourage drinking behaviour as we saw a reduction in motivation scores for the categories: social, enhancement and conformity. Our data showing that increased AUDIT scores are associated with increased anxiety, depression and ‘drinking to cope’ provides support to the prediction that pandemic-induced distress would increase alcohol consumption in some individuals whereas for others a reduction resulted from a decrease in physical and financial availability of alcohol.

Alcohol consumption increased in those respondents who reported increasing anxiety and depression levels and, most importantly, ‘drinking to cope’ with COVID-19 restrictions. This increase is of immense public health concern, given the consequences of problem drinking. Notably, the COLLATE study in Australia used similar assessment methods to identify individuals most likely to require support because of their increased drinking.^35^ We also found that individuals in the age range 41–50, a diagnosis of mental illness since the onset of the pandemic, currently working self-employed or furloughed staff, a confirmed positive COVID-19 diagnosis, smoking status and living in a residence without a garden were associated with increased AUDIT scores post-restrictions. Changes in alcohol consumption behaviours require further analysis as the pandemic progresses in order to evaluate any long-term consequences of this behavioural shift and further restrictions. Further understanding of alcohol consumption in these groups would help target prevention and treatment initiatives.

## Data Availability

The data are available from the corresponding author upon request.

## Supplementary material

For supplementary material accompanying this paper visit https://doi.org/10.1192/bjo.2021.986. click here to view supplementary material
