# Supplementary material for: Impact of COVID-19 restrictions on alcohol consumption behaviours
Source: BJPsych Open. 2021 Sep 14;7(5):e167. doi: 10.1192/bjo.2021.986 (PMC8446591; doi:10.1192/bjo.2021.986)
Supplement: Supplementary file 1 [file S2056472421009868sup001.docx]

# Supplementary Material

**Supplementary Material 1:** HANGOVER FREQUENCY QUESTIONNAIRE

Prior to any recommended restrictions due to COVID-19 how often did you have a drink containing alcohol?

Never

monthly or less

1-2 times a week

2-4 times a week

4 or more times a week

Prior to any recommended restrictions due to COVID-19 how many drinks containing alcohol did you have on a typical day when you were drinking?

1 or 2

3 or 4

5 or 6

7 to 9

10 or more

Prior to any recommended restrictions due to COVID-19 when was the last time you experienced a hangover?

In the last week

In the last 2 weeks

In the last month

In the last 6 months

In the last year

Prior to any recommended restrictions due to COVID-19 how frequently did you experience a hangover?

Never

monthly or less

1-2 times a week

2-4 times a week

or more times a week

Prior to any recommended restrictions due to COVID-19 how many drinks did you normally consume the night before you experienced a hangover?

1 or 2

3 or 4

5 or 6

7 to 9

10 or more

**Supplementary Material 2**: COVID Concern Score

Over the past 2 weeks, have you been more or less worried about the following?

Significantly less than usual 0

Slightly less than usual 1

Not more than usual 2

Slightly more than usual 3

Significantly more than usual 4

Marriage or other romantic relationship(s)

The wellbeing of your family

Work (even if you feel your job is safe)

Losing your job

Finances

Getting medication

Getting food

Your health

Your own safety/ security

Internet access

Boredom

Future plans

Supplementary Material 3. **Alcohol Use Disorder Identification Test (AUDIT) score comparison between the current study findings and the Adult Psychiatric Morbidity Survey (APMS) 2014 (17)**. AUDIT Category scoring from Public Health England Guidance (11) Low risk (AUDIT score 1-7), Hazardous drinking (AUDIT score 8-15), Harmful drinking/mild dependence (AUDIT score 16-19) and Probable dependence (AUDIT score 20+).

|  | *16-25* | | *26-30* | | *31-40* | | *41-50* | | *51-60* | | *60+* | | *All* | |
| --- | --- | --- | --- | --- | --- | --- | --- | --- | --- | --- | --- | --- | --- | --- |
|  | *%* | | *%* | | *%* | | *%* | | *%* | | *%* | | *%* | |
|  | *pre* | *post* | *pre* | *post* | *pre* | *post* | *pre* | *post* | *pre* | *post* | *pre* | *post* | *pre* | *post* |
| *Low risk* | *29* | *73* | *39* | *66* | *61* | *70* | *55* | *61* | *67* | *71* | *72* | *80* | *47* | *69* |
| *Hazardous drinking* | *52* | *21* | *46* | *26* | *30* | *20* | *26* | *13* | *21* | *21* | *16* | *16* | *38* | *21* |
| *Harmful drinking/mild dependence* | *13* | *2* | *11* | *5* | *5* | *3* | *11* | *11* | *13* | *8* | *4* | *0* | *10* | *4* |
| *Probable dependence* | *5* | *4* | *4* | *3* | *5* | *9* | *8* | *16* | *0* | *0* | *8* | *4* | *5* | *6* |
|  |  | |  | |  | |  | |  | |  | |  | |
|  | *16-24* | | *25-34* | | *35-44* | | *45-54* | | *55-64* | | *65+* | | *All* | |
|  | *%* | | *%* | | *%* | | *%* | | *%* | | *%* | | *%* | |
| ***APMS 2014 data (17)*** |  | |  | |  | |  | |  | |  | |  | |
| *Low risk* | *62* | | *70* | | *72* | | *77* | | *73* | | *88* | | *74* | |
| *Hazardous drinking* | *32* | | *25* | | *22* | | *20* | | *23* | | *11* | | *22* | |
| *Harmful drinking/mild dependence* | *4* | | *3* | | *3* | | *2* | | *2* | | *1* | | *2* | |
| *Probable dependence* | *2* | | *2* | | *3* | | *1* | | *1* | | *0* | | *2* | |

Supplementary Material 4. Implementation of COVID-19 restrictions. % of characteristic is shown for or each group (Increased vs Decreased). (A) % of Males and Females that increased or decreased alcohol consumption. (B) % of individuals with a history of Alcohol dependence that increased or decreased alcohol consumption. (C) % by age range of individuals that increased or decreased alcohol consumption
